# High-Fat-Diet-Induced Weight Gain Ameliorates Bone Loss without Exacerbating AβPP Processing and Cognition in Female APP/PS1 Mice

**DOI:** 10.3389/fncel.2014.00225

**Published:** 2014-08-08

**Authors:** Yunhua Peng, Jing Liu, Ying Tang, Jianshu Liu, Tingting Han, Shujun Han, Hua Li, Chen Hou, Jiankang Liu, Jiangang Long

**Affiliations:** ^1^Center for Mitochondrial Biology and Medicine, The Key Laboratory of Biomedical Information Engineering of Ministry of Education, School of Life Science and Technology and Frontier Institute of Science and Technology, Xi’an Jiaotong University, Xi’an, China; ^2^Shaanxi Translational Center for Functional Foods, Xi’an, China

**Keywords:** Alzheimer’s disease, osteoporosis, weight gain, bone mineral density, leptin

## Abstract

Osteoporosis is negatively correlated with body mass, whereas both osteoporosis and weight loss occur at higher incidence during the progression of Alzheimer’s disease (AD) than the age-matched non-dementia individuals. Given that there is no evidence that being overweight is associated with AD-type cognitive dysfunction, we hypothesized that moderate weight gain might have a protective effect on the bone loss in AD without exacerbating cognitive dysfunction. In this study, feeding a high-fat diet (HFD, 45% calorie from fat) to female APP/PS1 transgenic mice, an AD animal model, induced weight gain. The bone mineral density, microarchitecture, and biomechanical properties of the femurs were then evaluated. The results showed that the middle-aged female APP/PS1 transgenic mice were susceptible to osteoporosis of the femoral bones and that weight gain significantly enhanced bone mass and mechanical properties. Notably, HFD was not detrimental to brain insulin signaling and AβPP processing, as well as to exploration ability and working, learning, and memory performance of the transgenic mice measured by T maze and Morris water maze, compared with the mice fed a normal-fat diet (10% calorie from fat). In addition, the circulating levels of leptin but not estradiol were remarkably elevated in HFD-treated mice. These results suggest that a body weight gain induced by the HFD feeding regimen significantly improved bone mass in female APP/PS1 mice with no detriments to exploration ability and spatial memory, most likely via the action of elevated circulating leptin.

## Introduction

Alzheimer’s disease (AD), the most common type of dementia, is defined as “the disease or injury which initiated the train of events leading directly to death” by World Health Organization (Alzheimer’s Association, 2014). Growing incidence rate of AD worldwide makes it emergent to deal with its complications, such as osteoporosis (Bredesen and John, 2013). Some studies have indicated that low bone mineral density (BMD) or an increased rate of BMD loss is associated with a higher risk of AD (Tan et al., 2005; Zhou et al., 2011). Epidemiology studies also show that AD itself is an independent risk factor for bone impairment (Sato et al., 1998; Weller and Schatzker, 2004; Loskutova et al., 2009), particularly in postmenopausal females (Andersen et al., 1999; Yoshimura et al., 2005). More importantly, in patient with AD, the progression of osteoporosis is correlated with the AD pathogenesis (Lee et al., 2012).

It is evidenced that low body mass index (BMI) is associated with osteoporosis (Felson et al., 1993; Tremollieres et al., 1993; Ravn et al., 1999; De Laet et al., 2005), while obese individuals generally exhibit high BMD and bone strength (Reid et al., 1992; Khosla et al., 1996; Reid, 2002; Andersen et al., 2014; Johansson et al., 2014). Patients with AD exhibit a significant weight loss compared to the age-matched normal controls (White et al., 1996, 1998; Cronin-Stubbs et al., 1997; Stewart et al., 2005). There is no debate that being severely obese (BMI > 40) increases the risk for many illnesses and even increases mortality. However, the moderate overweight (25 < BMI < 30) showed a protective effect of lowering the mortality (Flegal et al., 2013; Couzin-Frankel, 2014; Solon-Biet et al., 2014), and some evidences suggest that being overweight is not associated with AD-type cognitive dysfunction (Kalmijn et al., 2000; Kivipelto et al., 2005). Thus, we hypothesized that moderate weight gain may improve the bone loss without exacerbating cognitive dysfunction in AD.

As one approach leading to weight gain, an extremely high-fat diet (HFD, 60% calorie from fat) was previously shown to induce a remarkable brain insulin resistance as well as spatial memory impairment in a normal mouse or a transgenic mouse model of AD (Ho et al., 2004; Moroz et al., 2008; Leboucher et al., 2013). In contrast, a moderate HFD (45% calorie from fat) caused little impairment in central insulin signaling and spatial memory (Mielke et al., 2006; McNeilly et al., 2012), although an operant bar-pressing task appeared to be capable of distinguishing the mice fed a moderate HFD from the vehicle controls (Mielke et al., 2006; McNeilly et al., 2011). Therefore, in this study, we induced a body weight gain in female APP/PS1 transgenic mice by feeding a moderate HFD (45% calorie from fat) to investigate the bone remodeling and the cognitive function. We found that severe femoral bone loss occurred in this transgenic mouse model of AD, and the body weight gain induced by the HFD greatly improved bone mass and mechanic properties. However, cerebral insulin signaling, AβPP processing, exploration ability, and spatial learning and memory were not deteriorated in transgenic mice fed HFD compared with the transgenic mice fed the control diet (10% calorie from fat). Our results suggested that the body weight management deserves to be evaluated in individuals with AD, especially those who experience progressive weight loss and osteoporosis.

## Materials and Methods

### Animals

The APPswe/PS1dE9 (APP/PS1) transgenic mice express both human presenilin 1 (A246E variant) and a chimeric amyloid precursor protein (APPswe) under direction of the mouse prion protein promoter, and on the C57BL/6J background (Borchelt et al., 1997). Three-month-old APP/PS1 female transgenic mice and non-transgenic littermates (C57BL/6 mice) were used for this study. The mice were purchased from Nanjing Biomedical Research Institute of Nanjing University (Nanjing, Jiangsu, China) and housed at 23–25°C with 60% humidity under 12:12 h light:dark cycles, with free access to food and water throughout the experiment. The mice were divided into three groups: wild-type mice (C57BL/6 group, *n* = 10), APP/PS1 transgenic mice (APP/PS1 group, *n* = 12), and APP/PS1 transgenic mice fed the HFD (APP/PS1 + HFD group, *n* = 11). During the 6 months of feeding, the mice consumed *ad libitum* either a control purified diet (10% calorie from fat) for the C57BL/6 group and APP/PS1 group, or an HFD (45% calorie from fat) for APP/PS1 + HFD group. The composition of each diet and the fatty acid profile of each diet are listed in Table S1 in Supplementary Material. The detailed formula of each fat diet is given in Table S2 in Supplementary Material. The body weights were recorded weekly. All the experimental procedures followed the Guide for the Care and Use of Laboratory Animals: Eighth Edition, ISBN-10:0-309-15396-4, and the animal protocol was approved by the animal ethics committee of School of Life Science, Xi’an Jiaotong University.

At 8.5 months of age, the mice were subjected to a T-shaped maze on 1 day. Three days later, the mice were subjected to a water maze every day for 7 days. The mice were sacrificed by decapitation 3 days after the end of the water maze testing, so that the age of mice when they were sacrificed was 9 months. The brain, liver, and serum were harvested, as well as the femurs in both hindlimbs. The left femur was stored in saline at 4°C for the measurement of mechanical parameters. The right femur was fixed in 10% formaldehyde for micro-CT scanning.

### Measurements of exploration ability, working, learning and memory performance with the T-maze and water maze

The T-shaped maze was constructed with one start arm and two goal arms, all with a length of 30 cm, width of 10 cm, and height of 20 cm. Spontaneous alternation of the T-maze is based on the assumption that the mouse always prefers to explore the new arm rather than the familiar arm (Gerlai, 1998). The tested mouse was first placed in the start arm of the T-maze. The mouse was then free to choose to enter either the left or the right goal arm (Reisel et al., 2002; Deacon and Rawlins, 2005). The choices of each mouse were recorded to calculate the percentage of alternations for eight trials with a 15-min interval between each trail. The latency time to reach the goal arm for each mouse was also recorded.

The Morris water maze tests were conducted in a diameter of 120 cm round tank filled with opaque water. The water was kept at 25°C and surrounded by dark walls containing geometric designs as visual cues. The hidden 7.5 diameter platform was submerged 0.5 cm below the water surface. The day before the first testing day, each mouse was allowed to swim freely in the tank for 90 s. On the first day of the training, each mouse was placed in two different quadrants of the pool and was required to find the hidden platform for two trials with a maximum of 120 s for each trial. For the next 5 days, each mouse was placed in three different quadrants of the pool and allowed a maximum of 120 s to find the submerged platform. During the consecutive 6 days of training, if the mice failed to find the platform within 120 s, they were physically guided to it and allowed to remain on the platform for 20 s. On the seventh day that after 6 days of training, the platform was removed and mice were tested with a probe trial. The total time for each mouse was 45 s. The performance in all tasks (escape latency to platform and distance to target quadrant during training phase; mean crossings on platform during probe trial) was recorded using a computer-based video tracking system (Vorhees and Williams, 2006).

### Measurement of brain Aβ

The cortex was dissected and homogenized in ice-cold guanidine buffer (5 M guanidine HCl/50 mM Tris HCl, pH 8.0) containing protease inhibitor mixture (5 mM sodium pyrophosphate, 1 mM β-glycerolphosphate, 1 mM Na_3_VO_4_, 1 mM EDTA, and 1 μg/ml leupetin). The amounts of soluble Aβ_40_ and Aβ_42_ in brain cortical homogenates were determined by ELISA kits for Aβ_40_ and Aβ_42_ (Invitrogen, Camarillo, CA, USA), respectively, according to the manufacturer’s instructions. In brief, 50 μl standards of known human Aβ_40_ or Aβ_42_ and the tested samples were added to the wells pre-coated with NH_2_-terminus of human Aβ. Then, 50 μl human Aβ_40_ or Aβ_42_ detection antibody solution was added into each well. The standards and samples were detected with horseradish peroxidase (HRP)-labeled anti-rabbit antibodies for the COOH-terminus of the 1–40 Aβ or 1–42 Aβ sequence. The reactions were stopped by adding 100 μl stop solution. The optical density (OD) at a wavelength of 450 nm was measured by FlexStation 3 microplate reader (Molecular Devices, Sunnyvale, CA, USA). The protein concentrations of the homogenates were determined using the BCA method, and Aβ_40_ and Aβ_42_ were expressed as pg Aβ_40_ or Aβ_42/_mg protein.

### Western blot

The brain samples were lysed with Western and IP lysis buffer (Beyotime, Haimen, Jiangsu, China) and centrifuged at 13,000 g for 15 min. The protein concentrations were determined using the BCA protein assay kit, and equal amounts of each protein (10 μg) were subjected to sodium dodecyl sulfate polyacrylamide gel electrophoresis (SDS-PAGE), transferred to pure nitrocellulose membranes (PerkinElmer Life Sciences, Boston, MA, USA), and blocked with 5% non-fat milk in Tris-buffered saline Tween-20 (TBST) buffer. The membranes were incubated with primary antibodies (diluted by 1:1,000 to 1:10,000) for 3 h at room temperature. The antibodies against AβPP, AKT, p-AKT, GSK3β, and p-GSK3β were purchased from Cell Signaling Technology (Danvers, MA, USA), the Aβ antibody anti-6E10 was from Covance (Dedham, MA, USA), and the antibody against actin was from Invitrogen (Carlsbad, CA, USA). The membranes were subsequently incubated with anti-rabbit or anti-mouse antibodies for 1 h at room temperature. Chemiluminescent detection was performed using the Pierce ECL Western blotting substrate, and the signals were analyzed using Quantity One software.

### Femur mechanical property testing

The mechanical properties of the femurs were determined using a three-point bending test that fractured the middle of the femoral region. The round-surfaced cross-head probe of a servo-controlled electromechanical testing system (Instron 5567, Canton, MA, USA) contacted the medial femur surface at its longitudinal midpoint within a 10 mm loading span. Loading speed was applied at 1 mm/min, and the load–deformation curve was recorded for calculation of elastic stress, elastic modulus, elastic force and ultimate force to fracture, energy absorption, and stiffness. The femur length was measured using digital calipers. The maximum and minimum outer and inner diameters of the fractured section were also measured using digital calipers for calculation of the second moment of inertia. The elastic force was the maximum load of the elastic (linear) region of the load–deformation curve. The ultimate force was the maximum load when the fracture occurred, which is defined as the height of the curve. Energy absorption was calculated as the product of load and displacement, defined as the area under the elastic region of the curve. Stiffness was calculated as the slope of the elastic region of the curve. The second moment of inertia (*J*), elastic stress (σ_φ_), and elastic modulus (*E*) were calculated according to the formulas below (Jämsä et al., 1998; Turner, 2002; Akhter et al., 2004; Bonnet et al., 2007; Yang et al., 2009).

$$\begin{array}{llll} J & =\frac{BH^{\text{3}}-bh^{\text{3}}}{\text{64}}\pi & & \;\\ \sigma_{\varphi } & =\frac{F_{\text{p}}-H\times L}{8\times J}\; & & \;\\ E & =\frac{F_{\text{p}}-L^{\text{3}}}{48\times d_{\text{p}}\times J}\; & & \; \end{array}$$

where *F*_p_ is the difference between the highest and lowest loads of the elastic part of the load–displacement curve; *d*_p_ is the difference between the maximum and minimum displacements of the elastic part of the load–displacement curve; *L* is the span length between two support points (10 mm); and *H, h, B*, and *b* are the maximum outer diameter, minimum outer diameter, maximum inner diameter, and minimum inner diameter of the fracture section, respectively.

### Micro-CT scanning

To determine the BMD and microarchitecture, the right femur was scanned using a micro-CT scanner (GE eXplore Locus SP Micro-CT, GE Healthcare, Barrington, IL, USA). All scans were performed with 80 kV tube voltage and 80 μA tube current and an exposure time of 3000 ms. The voxel size was 8.0 μm × 8.0 μm × 8.0 μm for the trabecule analysis, and the angle of the increment was set to 0.5°. A fixed threshold was used to extract the mineralized bone phase by averaging the critical value of the grayscale. The region of interest (ROI) was the entire volume inside the femur head. The trabecular volumetric BMD (vBMD), representing the apparent BMD of the trabecule at the organ level, was calculated from all voxels in the ROI. The trabecular tissue BMD (tBMD), representing the BMD at the tissue level, was defined as the tissue mineral content divided by the volume of the fixed threshold voxels.

Other data were also calculated from the ROI, including bone volume fraction (BV/TV), trabecular number (Tb.N), trabecular thickness (Tb.Th), and trabecular separation (Tb.Sp). For the cortical bone, the data were taken from a 2-mm-long round region of the mid-diaphysis femur and included the mean thickness (Ct.Th), bone mineral content (BMC) and density (BMD), total area (Tt.Ar), cortical area (Ct.Ar), marrow area (Ma.Ar), and cortical area fraction (Ct.Ar/Tt.Ar) (Bouxsein et al., 2010).

All the micro-CT data were calculated using MicroView v2.1.1 software and Advanced Bone Analysis application (GE Healthcare, Barrington, IL, USA).

### Biochemical assays of serum markers

The blood samples were allowed to clot undisturbed for 30 min at room temperature. The sera were separated from the blood samples by centrifugation at 1,500 rpm for 15 min. The serum total cholesterol (mmol/L), triglycerides (mmol/L), alkaline phosphatase (ALP, IU/L), calcium (Ca^2+^, mmol/L), and phosphorus (P, mmol/L) were measured using an automated HITACHI 7600 clinical analyzer (HITACHI, Ltd., Tokyo, Japan).

The serum estradiol levels were measured using a mouse estradiol (E2) ELISA Assay Kit according to the manual (Nanjing Jiancheng Bioengineering Institute, Nanjing, China). Briefly, 40 μl samples, 10 μl biotin-labeled mouse E2 antibodies, and 50 μl HRP-conjugated streptavidin were combined in this order in the assay plate wells, which were pre-coated with mouse E2 monoclonal antibody. After incubation for 60 min at 37°C, the plate was washed five times to remove the uncombined enzyme, and the substrate components A (50 μl) and B (50 μl) were added to the well. After approximately 15 min of reaction time, the reaction was stopped by adding 50 μl of the stop solution. The OD at 450 nm was measured using a FlexStation 3 microplate reader.

The serum leptin levels were measured using a mouse leptin ELISA kit (Crystal Chem, Downers Grove, IL, USA) according to the manufacturer’s instructions. Briefly, 45 μl of the sample diluent, 50 μl anti-mouse leptin serum, and 5 μl sample were added in this order to the assay plate wells, which were pre-coated with mouse leptin antibody. After incubation overnight at 4°C, the plate was washed five times to remove the uncombined enzyme, and 100 μl substrate was added to each well. After approximately 30 min at room temperature, the reaction was stopped by adding 100 μl stop solution. The ODs at 450 and 630 nm were measured using a FlexStation 3 microplate reader.

### Statistical analysis

Ten C57BL/6 mice, 12 APP/PS1 mice, and 11 APP/PS1 + HFD mice were fed since 3 months old. Thus, for the measurements of body weight, spatial learning and memory, and mechanical properties, the sample size was 10 for C57BL/6 mice, 12 for APP/PS1 mice, and 11 for APP/PS1 + HFD mice. When the mice were sacrificed, 3 mice from each group were randomly selected to perfuse paraformaldehyde for immunohistochemistry experiments (data not shown in the text). For ELISA and western blot, the sample size is 7 for C57BL/6 mice, 9 for APP/PS1 mice, and eight for APP/PS1 + HFD mice. Due to the fact that a femur in APP/PS1 group was damaged and excluded from samples, 7 of C57BL/6 mice, 8 of APP/PS1 mice, and 8 of APP/PS1 + HFD mice were used for the micro-CT scanning.

All data were expressed as the means ± SEM. One-way analysis of variance (ANOVA) with a Newman–Keuls *post hoc* test or two-way ANOVA with a Bonferroni’s *post hoc* test was used to determine the differences among the groups. Significance was defined at the 0.05 level.

## Results

### Body weight, perimetric fat weight, and levels of serum cholesterol and triglyceride

**Figure 1:**
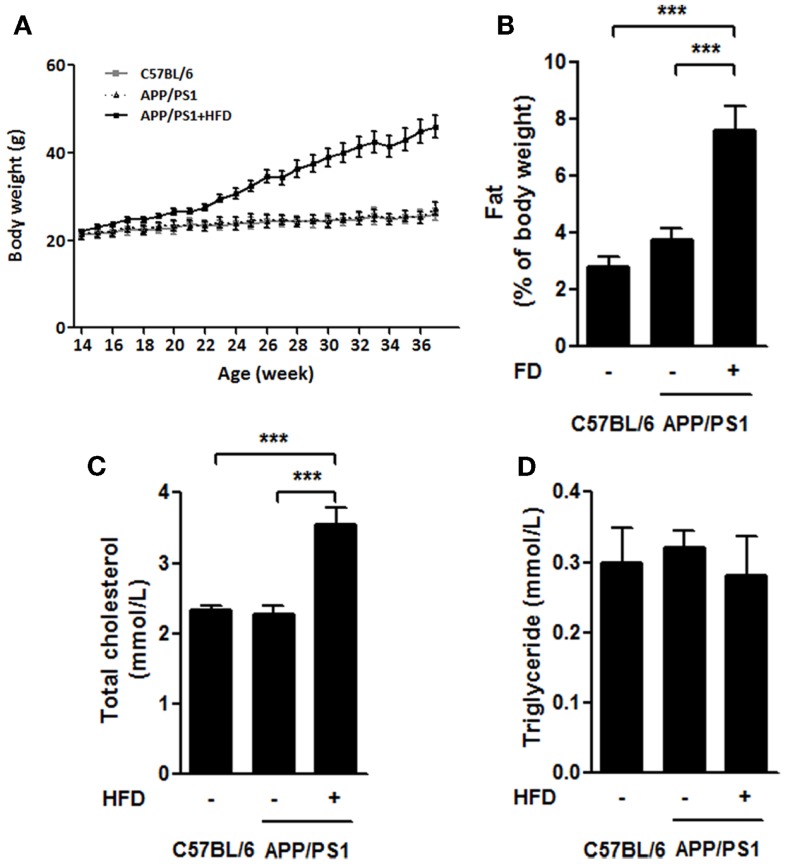
**High-fat-diet-induced weight gain in APP/PS1 mice**. Throughout the experiment, the body weights of C57BL/6 and APP/PS1 mice had no difference, and significant body weight gain was induced after HFD feeding for 8 weeks (at 22 weeks) and sustained growing from then on **(A)**. The ratio of parametric fat pad weight to body weight **(B)** of APP/PS1 + HFD mice was significantly higher than that of the C57BL/6 and APP/PS1 mice. The serum level of total cholesterol in APP/PS1 + HFD mice was higher than those in the other two mice groups **(C)**. No difference in serum triglycerides was found among the three groups **(D)**. Data were means ± SEM. *n* = 10 for C57BL/6 mice, *n* = 12 for APP/PS1 mice, and *n* = 11 for APP/PS1 + HFD mice for body weight. *n* = 7 for C57BL/6 mice, *n* = 9 for APP/PS1 mice, and *n* = 8 for APP/PS1 + HFD mice for other measurements. All results were analyzed by one-way ANOVA, followed by Newman–Keuls *post hoc* test, except that the body weight result was analyzed by two-way ANOVA, followed by Bonferroni’s *post hoc* test. ***p < 0.001.

During the feeding period, the initial body weights of the mice were 21.66 ± 1.29 g, and the final body weights of APP/PS1 + HFD mice (46.00 ± 9.72 g) were approximately 77.2 and 68.8% greater than those of C57BL/6 (25.96 ± 1.29 g) and APP/PS1 mice (27.25 ± 1.45 g), respectively, whereas no difference was found between the body weights of the C57BL/6 mice and APP/PS1 groups (Figure 1A). The ratio of the perimetric fat pad weight to body weight was 1.7 and 1.0 times higher in the APP/PS1 + HFD mice than those of the C57BL/6 and APP/PS1 mice, respectively (Figure 1B). A higher level of serum cholesterol was observed in the transgenic mice that were fed the HFD, while levels of serum triglycerides were not different among all groups (Figures 1C,D).

### Exploration ability and spatial learning and memory measured by T-maze and water maze

**Figure 2:**
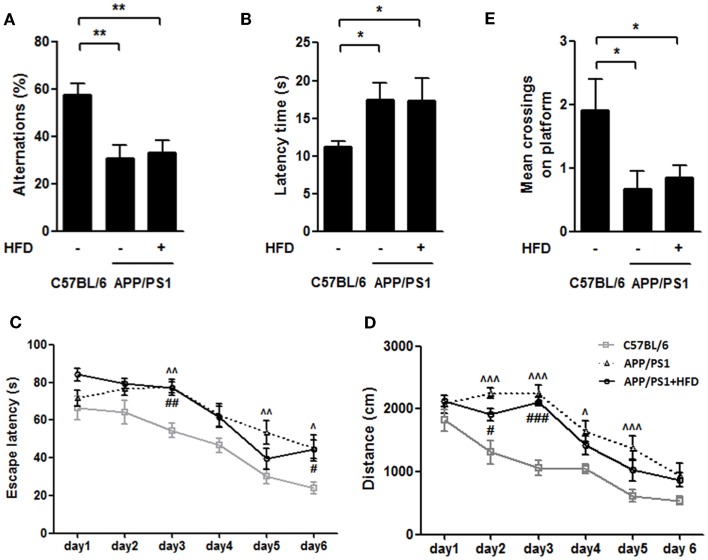
**HFD did not deteriorate the working, learning and memory performance in APP/PS1 mice**. In the T-maze spontaneous alternation test, both APP/PS1 group and APP/PS1 + HFD group showed fewer percentages to make the alternative choices **(A)** and had a longer latency time **(B)** than C57BL/6 group. APP/PS1 group exhibited significant longer escape latency on days 3, 5, and 6, and longer distance on days 2, 3, 4, and 5, while APP/PS1 + HFD group exhibited significant longer escape latency on days 3, and 6, and longer distance on days 2 and 3 **(C,D)**. The mean crossings of the C57BL/6 group were more frequent than those of APP/PS1 and APP/PS1 + HFD groups **(E)**. Additionally, no difference was found between the APP/PS1 and APP/PS1 + HFD groups. Data were means ± SEM. *n* = 10 for C57BL/6 mice, *n* = 12 for APP/PS1 mice, and *n* = 11 for APP/PS1 + HFD mice. T-maze results were analyzed by one-way ANOVA, followed by Newman–Keuls *post hoc* test. *p < 0.05; **p < 0.01. The water maze result was analyzed by two-way ANOVA, followed by Bonferroni’s *post hoc* test. ^∧^*p* < 0.05; ^∧∧^*p* < 0.01; ^∧∧∧^*p* < 0.001, APP/PS1 versus C57BL/6; ^#^*p* < 0.05; ^##^*p* < 0.01; ^###^*p* < 0.001, APP/PS1 + HFD versus C57BL/6.

To evaluate whether the HFD accelerated the development of AD, the behavioral tests (T-maze and water maze) were conducted in the 9-month-old animals. The APP/PS1 group and APP/PS1 + HFD group exhibited impaired exploration ability, spatial learning and memory abilities, as indicated by fewer alternative choices and longer latency in the T-maze test, and longer escape latency and distance during training phase, and fewer crossings on the platform during probe trial in the water maze test compared to the C57BL/6 mice. Notably, no significant behavioral differences were found between the APP/PS1 and APP/PS1 + HFD groups (Figures 2A–E) in this study, suggesting that feeding a diet at a level of 45% calorie from fat for as long as 6 months does not lead to a detriment of exploration ability, spatial learning and memory in the female transgenic mouse model of AD.

### Brain insulin signaling and AβPP processing

**Figure 3:**
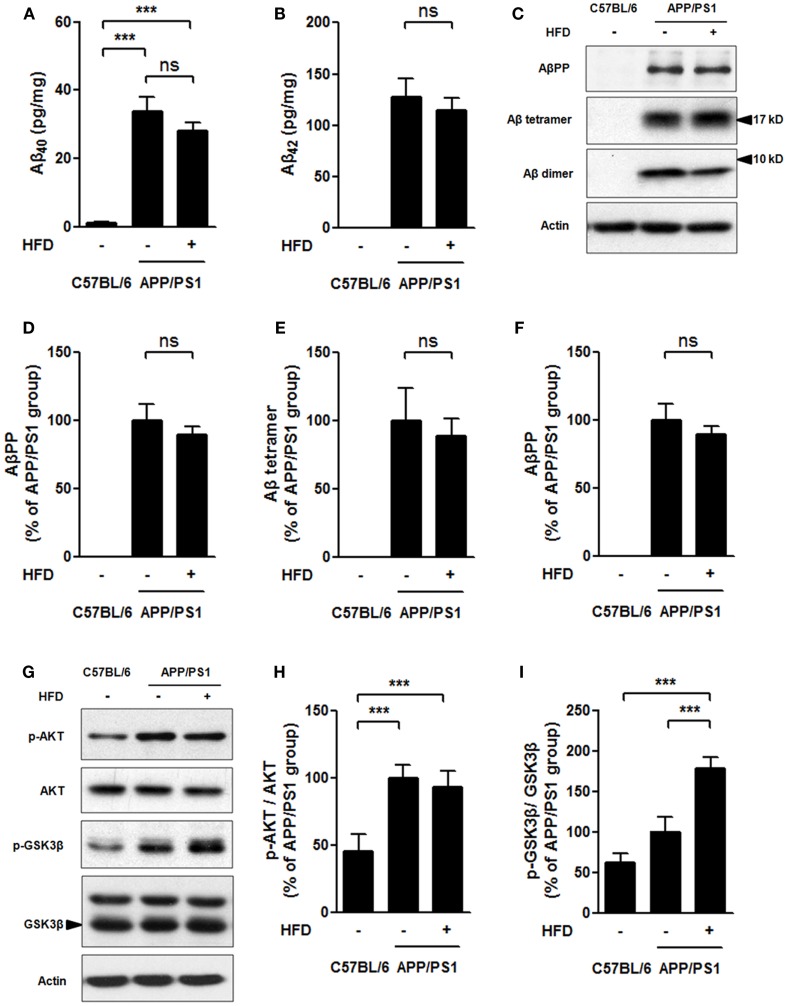
**Soluble Aβ level and brain insulin signaling**. Aβ_40_ **(A)**, and Aβ_42_ **(B)** levels were much higher in APP/PS1 and APP/PS1 + HFD mice compared with that those of C57BL/6 mice, and the Aβ_40_ and Aβ_42_ levels were determined to be the same between APP/PS1 and APP/PS1 + HFD mice by ELISA. The expression of AβPP, Aβ tetramer, Aβ dimer **(C)**, phosphorylation of AKT, and phosphorylation of GSK3β **(G)** in brain tissue were analyzed by western blot. Quantification of western blot of AβPP **(D)**, Aβ tetramer **(E)**, Aβ dimer **(F)**, p-AKT **(H)**, and p-GSK3β **(I)**. Data were means ± SEM. *n* = 7 for C57BL/6 mice, *n* = 9 for APP/PS1 mice, and *n* = 8 for APP/PS1 + HFD mice. The results were analyzed by one-way ANOVA, followed by Newman–Keuls *post hoc* test. ***p < 0.001.

Neuronal insulin resistance has been confirmed in the brain of AD (Talbot et al., 2012). The neuronal insulin acts through a similar tyrosine kinase pathway as peripheral insulin acts. Insulin binds to insulin receptor (IR) followed by phosphorylation of phosphatidylinositol 3-kinase (PI3K), which leads to AKT/protein kinase B (PKB) phosphorylation and activation, and then glycogen synthesis kinase 3 (GSK3) is phosphorylated and inactivated (El Khoury et al., 2014). HFD feeding is linked to cognitive impairment mediated by brain insulin resistance and accelerated Aβ generation (Ho et al., 2004; McNeilly et al., 2011). The neural levels of p-AKT and p-GSK3 were determined in this study to evaluate the brain insulin signaling. The Aβ levels were also measured in APP/PS1 mice fed HFD (Figures 3A–I).

In C57BL/6 mice, soluble Aβ_40_ and Aβ_42_ levels measured by ELISA, and Aβ precursor protein (AβPP), Aβ tetramer, and Aβ dimer levels assessed by western blots turned out to be very low or zero, whereas APP/PS1 and APP/PS1 + HFD mice had much higher levels of those parameters (Figures 3A–F). Importantly, the Aβ_40_, Aβ_42_, AβPP, Aβ tetramer, and Aβ dimer levels were comparable in the APP/PS1 + HFD mice and the APP/PS1 mice fed the normal-fat diet (Figures 3A–F). Consistent with these observations, the APP/PS1 and APP/PS1 + HFD mice showed elevated levels of AKT phosphorylation compared to the C57BL/6 mice, but no significant differences between APP/PS1 and APP/PS1 + HFD mice were observed (Figures 3G,H). The p-GSK3β expression was even higher in APP/PS1 + HFD mice than in APP/PS1 and C57BL/6 mice (Figures 3G,I). These results suggested that HFD did not aggravate Aβ generation and brain insulin signaling impairment in the female transgenic mice.

### Mechanical parameters of the femoral bone

**Figure 4:**
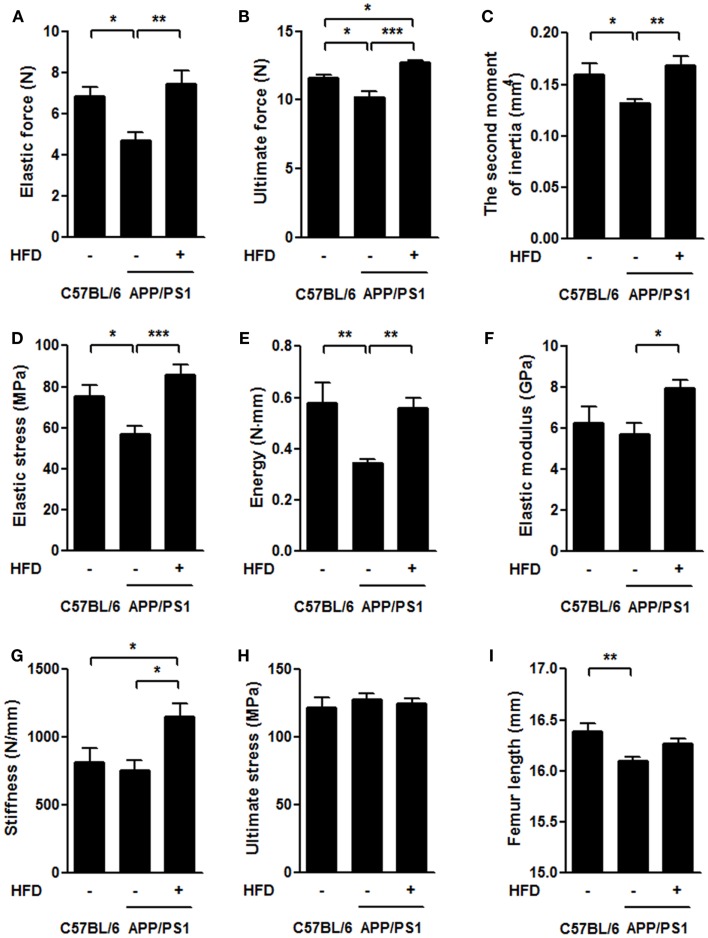
**HFD ameliorated impairments of femoral mechanical properties in APP/PS1 mice**. In the three-point bending test, the elastic force **(A)**, ultimate force **(B)**, second moment of inertia **(C)**, elastic stress **(D)**, and energy **(E)** were all significantly reduced in APP/PS1 mice compared to the age-matched C57BL/6 mice. No difference was found in elastic modulus **(F)** and stiffness **(G)** between C57BL/6 and APP/PS1 mice, but HFD significantly increased these two parameters. Ultimate stress did not vary among the three groups **(H)**. Femur length was shorter in APP/PS1 mice but not reversed by HFD **(I)**. Data were means ± SEM. *n* = 10 for C57BL/6 mice, *n* = 12 for APP/PS1 mice, and *n* = 11 for APP/PS1 + HFD mice. The results were analyzed by one-way ANOVA, followed by Newman–Keuls *post hoc* test. *p < 0.05; **p < 0.01; ***p < 0.001.

To understand the mechanical effects of AD pathology and HFD-induced body weight gain on bone, we employed the three-point bending test to measure the mechanical properties of the femurs (Figures 4A–H). We found that the elastic force and ultimate force were both significantly reduced in APP/PS1 mice compared to the age-matched C57BL/6 mice and that these effects were completely reversed by the HFD (Figures 4A,B). With respect to the second moment of inertia, which reflects the geometric size of bone, and load-displacement curve, we found that elastic stress and energy absorption decreased in the APP/PS1 mice and were reversed by the HFD (Figures 4C–E). Although no differences in the elastic modulus and stiffness were observed between the C57BL/6 and APP/PS1 mice, HFD significantly increased the values of these two parameters (Figures 4F,G). Ultimate stress was not different among the three groups of mice (Figure 4H). The femur length was shorter in APP/PS1 mice compared to C57BL/6 mice, and this effect was not altered by the HFD (Figure 4I).

### Bone mineral density and microarchitectural parameters of the femoral bone

**Figure 5:**
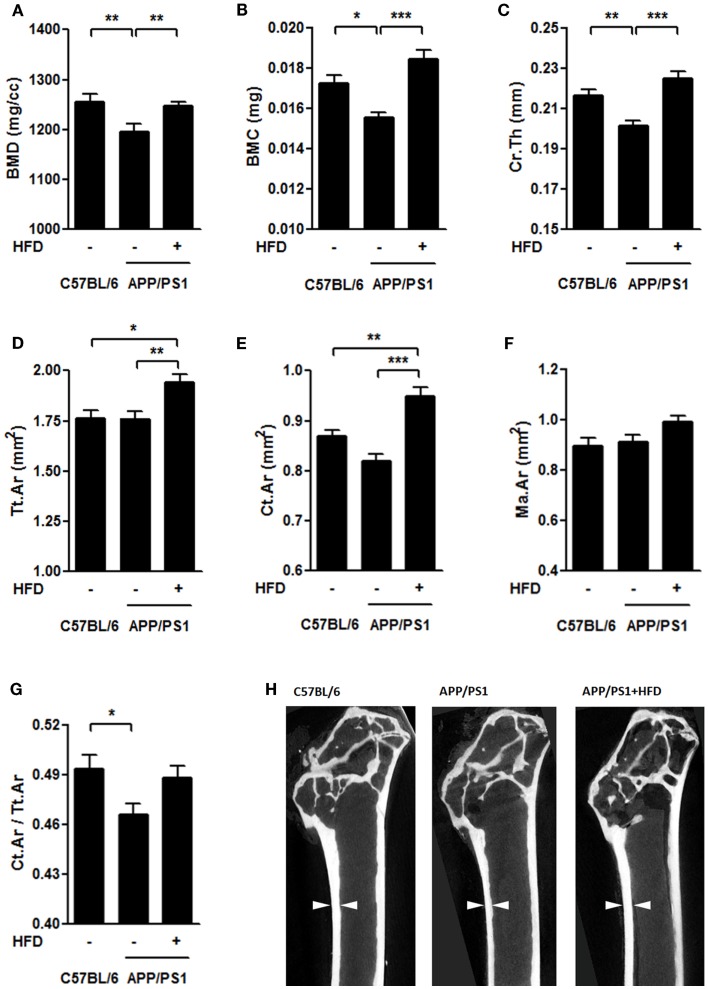
**HFD protected femoral cortical bone from AD-involved impairments**. The bone mineral density [BMD, **(A)**], bone mineral content [BMC, **(B)**], and mean thickness [Cr.Th **(C)**] were lower in the APP/PS1 mice compared to the C57BL/6 mice, all of which were reversed by HFD feeding. No significant decrease was found in total area [Tt.Ar **(D)**] and cortical area [Ct.Ar **(E)**] in the APP/PS1 mice compared to the C57BL/6 mice, whereas, both of which were elevated by HFD feeding.Marrow area (Ma.Ar) did not vary among the three groups **(F)**. The ratio of Ct.Ar to Tt.Ar was decreased in APP/PS1 mice **(G)**, but not reversed by HFD feeding. Micrographs showed the differences of thickness and inner bone surface among all the groups with arrows indicating the representative part **(H)**. Data were means ± SEM. *n* = 7 for C57BL/6 mice, *n* = 8 for APP/PS1 mice, and *n* = 8 for APP/PS1 + HFD mice. The results were analyzed by one-way ANOVA, followed by Newman–Keuls *post hoc* test. *p < 0.05; **p < 0.01; ***p < 0.001.

To determine the structural basis for the alteration in biomechanical properties, we further evaluated the cortical bone in femurs using a micro-CT system. Compared to the C57BL/6 group, the APP/PS1 group had lower values for BMD, BMC, and mean thickness (Ct.Th) of cortical femur but showed no significant decreases in total area (Tt.Ar) and cortical area (Ct.Ar) (Figures 5A–E,H). Ct.Ar/Tt.Ar was decreased in the APP/PS1 mice (Figure 5G) compared with the C57BL/6 mice. HFD feeding resulted in significant increases in BMD, BMC, Ct.Th, Tt.Ar, and Ct.Ar of the measured bone in APP/PS1 mice (Figures 5A–E,H). There were no significant differences in marrow area (Ma.Ar) among the groups (Figure 5F). The micrographs also revealed that the inner bone surface facing the marrow was much smoother in the C57BL/6 and APP/PS1 + HFD groups than in APP/PS1 group (Figure 5H).

**Figure 6:**
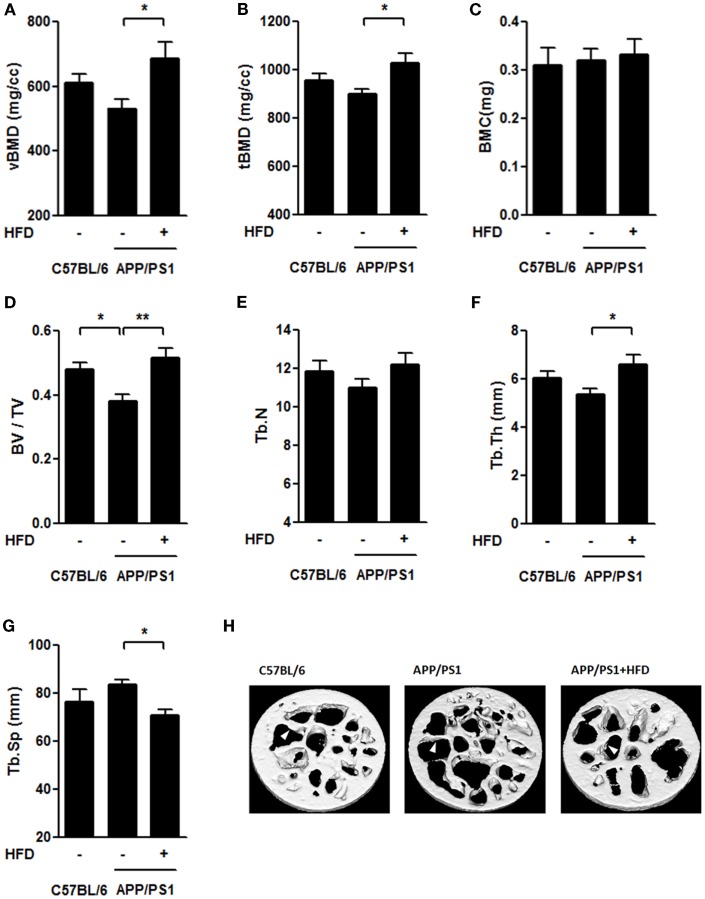
**HFD improved microarchitecture of femur trabecular bone in APP/PS1 mice**. The volumetric BMD [vBMD **(A)**], tissue BMD [tBMD **(B)**], and trabecular thickness [Tb.Th **(F)**] were not significantly altered in C57BL/6 mice and APP/PS1 mice; however, these parameters significantly increased after HFD. Bone volume fraction [BV/TV **(D)**] was lower in APP/PS1 mice and recovered after HFD feeding. No significant differences in BMC **(C)** or trabecular number [Tb. N **(E)**] were found among the three groups. The trabecular separation [Tb.Sp **(G)**] was not significantly altered in C57BL/6 mice or APP/PS1 mice, and it was reduced after HFD. Micrographs showed the microarchitecture of femoral trabecule **(H)** with arrows indicating the representative part. Data were means ± SEM. *n* = 7 for C57BL/6 mice, *n* = 8 for APP/PS1 mice, and *n* = 8 for APP/PS1 + HFD mice. The results were analyzed by one-way ANOVA, followed by Newman–Keuls *post hoc* test. *p < 0.05; **p < 0.01.

The femoral trabecular bone mass was also evaluated by micro-CT (Figures 6A–H). We found that the vBMD, tBMD, and trabecular thickness (Tb.Th) were the same in APP/PS1 mice and C57BL/6 mice, but these parameters were all significantly increased by the HFD feeding (Figures 6A,B,F,H). The trabecular separation (Tb.Sp) was similar in the APP/PS1 mice compared with the C57BL/6 mice but was reduced in mice on the HFD (Figure 6G). No significant differences in the BMC and trabecular number (Tb.N) were found among all groups (Figures 6C,E). The bone volume fraction (BV/TV) was lower in APP/PS1 mice than in the C57BL/6 mice and was corrected by the HFD feeding (Figure 6D).

### Serum alkaline phosphatase, calcium, phosphorus, estrogen and leptin levels

**Figure 7:**
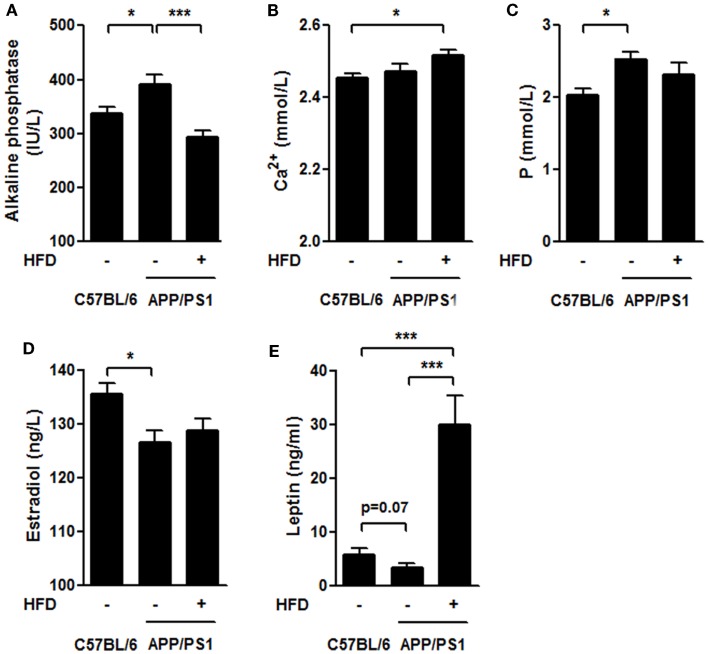
**Serum biochemical characteristics**. The ALP level was higher in APP/PS1 mice than that in C57BL/6 mice, and it was reversed after HFD feeding **(A)**. The serum Ca^2+^ levels in APP/PS1 mice was not different from that of C57BL/6 or APP/PS1 + HFD mice; however, it was higher in APP/PS1 + HFD mice than that in C57BL/6 mice **(B)**. The serum phosphorus level was higher in APP/PS1 mice than that in C57BL/6 mice, which was not reversed by HFD feeding **(C)**. APP/PS1 mice had a lower estrogen level than C57BL/6 mice, which was also not reversed by HFD feeding **(D)**. The serum leptin level was lower in APP/PS1 mice compared to C57BL/6 mice (*p* = 0.07), and it was significantly induced after HFD feeding **(E)**. Data were means ± SEM. *n* = 7 for C57BL/6 mice, *n* = 9 for APP/PS1 mice, and *n* = 8 for APP/PS1 + HFD mice. The results were analyzed by one-way ANOVA, followed by Newman–Keuls *post hoc* test. *p < 0.05; ***p < 0.001.

Several bone-remodeling-related markers, including serum ALP, calcium (Ca^2+^), phosphorus (P), circulating leptin and estradiol (Figures 7A–E), were measured in this study. The ALP activity was much higher in the APP/PS1 mice than in the C57BL/6 mice and this increase was significantly reversed by HFD feeding (Figure 7A). We found no significant difference in Ca^2+^ levels either between APP/PS1 mice and C57BL/6 mice, or between APP/PS1 mice and APP/PS1 + HFD mice, but Ca^2+^ levels were elevated in the APP/PS1 + HFD group compared with that in the C57BL/6 group (Figure 7B). The P concentration was higher in the APP/PS1 mice than in the C57BL/6 group, but this effect was unchanged by HFD feeding (Figure 7C). We found a lower concentration of estrogen in the APP/PS1 mice relative to the C57BL/6 mice, and the low level remained in the HFD-fed mice (Figure 7D). In contrast, there was a trend toward decreased levels of leptin in the APP/PS1 mice (*p* = 0.07) compared with the C57BL/6 mice, and this parameter was significantly elevated by the HFD feeding (Figure 7E).

## Discussion

### Severe bone loss and impaired biomechanical properties of the femur in female APP/PS1 transgenic mice

In APP/PS1 mice, the amyloid pathology can be observed in mice as young as 3 months, and the amyloid plaque is widely spread by middle age (9 to 12 months old) (Garcia-Alloza et al., 2006). In an earlier study by Yang et al., the measurement of BMD and microarchitecture parameters of both C57BL/6 mice and APP/PS1 mice was performed at the age of 6, 9, and 12 months, respectively. They found that, even though the amyloid pathology occurred as early as 3-month age in APP/PS1 mice, the deviation of BMD and microarchitecture could not be found until 9 months old. And at a later time point (12-month age), the deviation of BMD and microarchitecture become more obvious between AD and control mice. Therefore, taking the factors into consideration – amyloid pathology (as early as 3-month old) and osteoporosis pathology (at 9-month old), we evaluated the bone remodeling induced by weight gain during the period from AD pathogenesis to the typical pathology development, i.e., AD mice were given an HFD at 3 months of age and this lasted for 6 months, and the mice were then sacrificed at 9 months for all the measurements. By measuring the bone mechanical properties with the three-point bending test and measuring the BMD and microarchitecture with the micro-CT system, severe bone loss was detected in the femoral cortical bone of the transgenic mouse model of AD, suggesting that bone loss may be associated with amyloid pathogenesis (Yang et al., 2011; Xia et al., 2013).

As a result of the bone loss, the biomechanical properties of the femur were severely impaired in the AD mice compared with the age-matched C57BL/6 mice (Figure 4). These bone impairments were consistent with the serum biochemical indexes, including elevated serum ALP levels and P levels in the transgenic mice compared with the C57BL/6 mice (Figure 7). The results suggest a strong link between AD pathology and femoral bone impairment. In addition, we did not find the weight loss in middle-aged female AD mice in this study, because it is supposed to emerge in the later stage of the AD with more severe bone loss.

Yang et al. (2011) reported that in 9-month-old APP/PS1 mice, parameters including vBMD, tBMD, BV/TV, Tb.Th, and Tb.Sp in the proximal metaphysis of the tibiae differed from those in C57BL/6 mice. In this study, the femoral trabecule of the APP/PS1 mice showed no significant alternations of these parameters compared with the control mice (Figure 6). The differences of bone mass between tibiae and femoral trabecule in APP/PS1 mice may be due to the presence of fewer trabecule in the femur than that in the tibia. This finding is consistent with the observation that the femur is more resistant to bone loss than tibia (Lelovas et al., 2008).

### Moderate weight gain induced by HFD benefits AD-involved bone loss with no detriments of exploration ability and spatial learning and memory

In patients with AD, weight loss and osteoporosis are observed and become worse with AD progression (White et al., 1996; Cronin-Stubbs et al., 1997; Tan et al., 2005; Lelovas et al., 2008; Zhou et al., 2011), and the relative risk of death is positively correlated with increased weight loss in AD cases (White et al., 1998). In contrast to the weight loss, extreme obesity is a definite risk factor for many diseases, such as cardiovascular disease and cancer. A new meta-analysis revealed that obese people, particularly those who are extremely obese, tend to die earlier than those of normal weight. However, the findings also suggest that people who are overweight (but not obese) may live longer than people with clinically normal body weight (Flegal et al., 2013; Couzin-Frankel, 2014; Solon-Biet et al., 2014). A lot of studies reported that osteoporosis is accompanied with weight loss and bone mass can be improved by weight gain (Felson et al., 1993; Tremollieres et al., 1993; Frederich et al., 1995; Considine et al., 1996; Ravn et al., 1999; Lin et al., 2000; De Laet et al., 2005; Sheu and Cauley, 2011; Greco et al., 2013; Johansson et al., 2014). As expected in this study, the lower values of BMD and mechanical properties of the femur in transgenic mice were greatly improved by weight gain.

Notably, the benefit of HFD on bone health is specific for AD mice but not for normal C57 mice. In a previous study by Ionova-Martin et al. (2011), 15-week-old C57BL/6 mice received 16-week duration of HFD. Ultimate stress and ultimate force were 15% and 22% less in HFD-fed C57BL/6 mice compared with those in normal diet-fed C57BL/6 mice, respectively, while BMD, length, diameter, elastic modulus, and yield stress of their femora were comparable with those in normal diet-fed C57BL/6 mice. In another report by Shen et al. (2013), 3-month-old rats were given an 8-month duration of HFD. They found that HFD feeding increased bone formation and erosion rates, and decreased trabecular thickness at the tibia. BMD, Tb.N, and Tb.Th of femur in these HFD-fed rats were comparable with those in normal diet-fed rats. These studies clearly demonstrated that HFD feeding slightly influenced the mechanical properties and microarchitecture of bone in rodents.

The femoral bone benefits were fully demonstrated in this study; however, there is particular concern that body weight gain is generally linked to cognitive detriments. In this study, we avoided the extreme HFD (60% calorie from fat) feeding, which is known to lead to severe obesity, brain insulin resistance, and spatial memory impairment both in AD mouse model and normal C57BL/6 mouse (Ho et al., 2004; Moroz et al., 2008; Barron et al., 2013; Leboucher et al., 2013). Instead, we employed a 6-month feeding regimen of a 45% fat diet which resulted in a body weight gain in the transgenic mouse model of AD. The 6-month HFD feeding was implemented in 3-month-old APP/PS1 mice. Body weight at 5, 7, and 9 months (the end of the feeding) of HFD-fed mice were 17.9, 60.1, and 68.8% higher than the age-matched APP/PS1 mice fed with the normal-fat diet, respectively. These levels of weight gain are equivalent to a range of overweight to moderate obesity, but not severe obesity in humans. 45% HFD feeding, as reported in previous study, caused little impairment in the rodent central insulin signaling and spatial memory (Mielke et al., 2006; McNeilly et al., 2012) but a disability in performance of an operant bar-pressing task (Mielke et al., 2006; McNeilly et al., 2011). We confirmed that HFD did not exacerbate the exploration ability and working, learning, and memory performance during the 6 months of feeding compared to the transgenic mice fed a normal-fat diet (Figures 2A–D). Furthermore, the brain Aβ processing and brain insulin signaling, which are considered to be involved in Aβ plaque deposit and cognition dysfunction (Farooki, 2009), were not significantly altered by the HFD feeding (Figures 3A–I). Therefore, a strategy of moderate body weight gain, such as by the mean of moderate HFD regimen, should not be precluded, especially in case of diseases with progressive weight loss such as AD and AD-involved osteoporosis.

In addition to the effects of diets at different fat levels (10 or 45% calorie from fat) on spatial working, learning, and memory measurement as in this study, future studies should evaluate whether longer periods of HFD feeding lead to disability in other aspects of cognitive performance, such as delayed matching and non-matching to position task. Additionally, it remains to be defined whether the fat or the muscle gain is responsible for the bone improvement during HFD feeding. And other metabolic disorders will also need to be carefully assessed in future studies.

### Elevated circulating leptin induced by weight gain was involved in bone remodeling in female APP/PS1 mouse fed HFD

Both estrogen and leptin exhibit regulatory effects on bone formation and degradation. Leptin can ameliorate OVX-induced osteoporosis (Burguera et al., 2001). These effects may be due to peripheral stimulation of osteoblast proliferation and inhibition of osteoclastogenesis mediated by signaling through osteoprotegerin (OPG)/receptor–activator of NF-κB (RANK) and its ligand (RANKL). However, an indirect action of leptin via estradiol cannot be excluded (Yasuda et al., 1998; Thomas et al., 1999; Burguera et al., 2001; Holloway et al., 2002; Martin and Sims, 2005; Wada et al., 2006; Xue et al., 2012).

In the APP/PS1 mice, we found that the serum estradiol levels were reduced compared with the C57BL/6 mice. HFD feeding did not alter the circulating level of estradiol in the transgenic mice (Figure 7D), implying that estrogen level may contribute to bone loss but is not associated with the body weight gain-induced improvement of the bone mass in the transgenic mice. This finding is consistent with a report that total estrogen was similar in both obese and non-obese postmenopausal women (Albala et al., 1996).

Previous reports have well addressed the role of leptin in the regulation of bone health showing that the administration of leptin leads to an increase in the BMD in mice (Burguera et al., 2001; Sienkiewicz et al., 2011). In the AD brain, there are higher levels of leptin but lower levels of leptin receptor mRNA, thus suggesting that the AD brain exhibits impaired brain leptin signaling, namely less leptin receptor, as well as less interaction between the leptin and leptin receptors (Lieb et al., 2009; Zhou et al., 2011; Bonda et al., 2013). The enhanced bone mass induced by weight gain in female APP/PS1 mice might therefore be attributed to the peripheral action of leptin rather than brain leptin signaling (Burguera et al., 2001), through peripheral stimulation of osteoblast proliferation and inhibition of osteoclastogenesis (Holloway et al., 2002; Martin and Sims, 2005). In contrast to the HFD-induced beneficial effect to the bone quality in AD mice, a higher level of leptin induced by HFD in adult C57BL/6 mice did not lead to enhanced bone mechanical properties (Ionova-Martin et al., 2011), which suggests that leptin acts differently in osteoporosis-involved AD mice than it does in normal controls fed with HFD. Here, in this study, we still lack solid evidence linking leptin to bone quality in AD mice. Therefore, the underlying mechanism of leptin in regulating osteoblast proliferation and osteoclastogenesis still needs to be elucidated in female patient with AD.

Taken together, the body weight gain induced by the HFD feeding regimen completely improved the AD-involved femoral bone loss without adverse effects on the exploration ability and spatial memory impairment in the APP/PS1 transgenic mouse model of AD. Patients with AD always develop both progressive weight loss and osteoporosis. Our findings suggest that HFD may be an effective dietary administration for progressive weight loss and osteoporosis in patients with AD without a substantial impact on cognitive function. Further research is required to optimize the composition of the HFD in order to minimize the possible negative effect induced by HFD, for instance, the risk of cardiovascular disease and cognition impairment.

## Author Contributions

Yunhua Peng, Jing Liu, Jiangang Long, and Jiankang Liu contributed to the study conception and design. Yunhua Peng, Jing Liu, Hua Li, Chen Hou, and Ying Tang performed the experiments and collected data. Yunhua Peng and Jing Liu performed data analysis. Data were interpreted by Yunhua Peng, Jing Liu, Jianshu Liu, Tingting Han, Jiangang Long, and Jiankang Liu. Yunhua Peng, Jiangang Long, and Jiankang Liu drafted the manuscript. All authors critically revised the content of the manuscript and approved its final version. Jiangang Long and Jiankang Liu take responsibility for the integrity of the data analysis.

## Conflict of Interest Statement

The authors declare that the research was conducted in the absence of any commercial or financial relationships that could be construed as a potential conflict of interest.

## Supplementary Material

The Supplementary Material for this article can be found online at http://www.frontiersin.org/Journal/10.3389/fncel.2014.00225/abstract
