# Supplementary material for: High-Fat-Diet-Induced Weight Gain Ameliorates Bone Loss without Exacerbating AβPP Processing and Cognition in Female APP/PS1 Mice
Source: Front Cell Neurosci. 2014 Aug 8;8:225. doi: 10.3389/fncel.2014.00225 (PMC4125950; doi:10.3389/fncel.2014.00225)
Supplement: Supplementary file 1 [file Data_Sheet1.DOCX]

**Supplementary table 1. The constitution of the diets**

|  | **Constitution** | **Normal diet (%)** | **High fat diet (%)** |
| --- | --- | --- | --- |
| **Composition** | Fat | 10 | 45 |
|  | Protein | 20 | 19 |
|  | Carbohydrate | 70 | 36 |
| **Fatty acid profile** | Fat | 10 | 45 |
|  | Protein | 20 | 19 |
|  | Carbohydrate | 70 | 36 |
