# Supplementary material for: High-Fat-Diet-Induced Weight Gain Ameliorates Bone Loss without Exacerbating AβPP Processing and Cognition in Female APP/PS1 Mice
Source: Front Cell Neurosci. 2014 Aug 8;8:225. doi: 10.3389/fncel.2014.00225 (PMC4125950; doi:10.3389/fncel.2014.00225)
Supplement: Supplementary file 2 [file Data_Sheet2.DOCX]

**Supplementary table 2. The detailed formula of the diets**

| **Formula** | **Normal diet - 10% calorie from fat (g/Kg)** | **High fat diet - 45% calorie from fat (g/Kg)** |
| --- | --- | --- |
| Casein | 210 | 245 |
| L-Cystine | 3 | 3 |
| Corn Starch | 280 | 85 |
| Maltodextrin | 50 | 115 |
| Sucrose | 325 | 200 |
| Lard | 20 | 195 |
| Soybean Oil | 20 | 30 |
| Cellulose | 37 | 58 |
| Mineral Mix | 35 | 43 |
| Calcium Phosphate, dibasic | 2 | 3 |
| Vitamin Mix | 15 | 19 |
| Choline Bitartrate | 3 | 3 |
